# Increasing incidence of adult idiopathic inflammatory myopathies in the City of Salford, UK: a 10-year epidemiological study

**DOI:** 10.1093/rap/rky035

**Published:** 2018-09-17

**Authors:** Matthew J S Parker, Alexander Oldroyd, Mark E Roberts, William E Ollier, Robert P New, Robert G Cooper, Hector Chinoy

**Affiliations:** 1 Rheumatology Department, Salford Royal NHS Foundation Trust, Manchester Academic Health Science Centre, Salford; 2 NIHR Manchester Biomedical Research Centre, Manchester University NHS Foundation Trust, Manchester Academic Health Science Centre, The University of Manchester; 3 Arthritis Research UK Centre for Epidemiology, Centre for Musculoskeletal Research, University of Manchester, Manchester Academic Health Science Centre, Manchester; 4 Department of Neurology, Greater Manchester Neurosciences Centre, Salford Royal NHS Foundation Trust, Manchester Academic Health Science Centre, Salford; 5 MRC-ARUK Centre for Integrated Research into Musculoskeletal Ageing, University of Liverpool, Liverpool; 6 Centre for Integrated Genomic Medical Research, Division of Population Health, Health Services Research and Primary Care, Faculty of Biology, Medicine and Health, Manchester Academic Health Science Centre, The University of Manchester, Manchester, UK

**Keywords:** myositis, inflammatory muscle diseases, inflammatory myopathy, PM, DM, myositis, inclusion bodies, classification

## Abstract

**Objectives:**

The aim was to identify and characterize all incident adult cases of idiopathic inflammatory myopathies (IIM) between 1 January 2007 and 31 December 2016 in the City of Salford, UK.

**Methods:**

Adults first diagnosed with IIM within the study period were identified by: a Salford Royal NHS Foundation Trust (SRFT) inpatient episode IIM-specific ICD-10 coding search; all new patient appointments to SRFT neuromuscular outpatient clinics; and all Salford residents enrolled within the UKMYONET study. All patients with definite IIM by the 2017 EULAR/ACR classification criteria were included, as were probable cases if consensus expert opinion agreed. Cases were excluded if <18 years of age at disease onset, if they did not meet probable criteria or when probable but expert opinion concluded a non-IIM diagnosis.

**Results:**

The multimodal case ascertainment identified 1156 cases which, after review and application of exclusion criteria, resulted in 32 incident cases during the study period. Twenty-three of 32 were female, with a mean age of 58.1 years. The mean incidence of adult IIM was 17.6/1 000 000 person years, and higher for females than for males (25.2 *vs* 10.0/1 000 000 person years, respectively). A significant incidence increase over time was apparent (13.6 *vs* 21.4/1 000 000 person years; *P* = 0.032). Using EULAR/ACR classification criteria, the largest IIM subtype (21/32) was PM, followed by DM (8/32), IBM (2/32) and amyopathic DM (1/32). Expert opinion subtype differed from EULAR/ACR classification criteria in 19/32 cases.

**Conclusion:**

The incidence of adult IIM in Salford is 17.6/1 000 000 person years, higher in females, and is increasing over time. Disagreement exists between EULAR/ACR-derived and expert opinion-derived IIM subtype assignments.

**Key messages**

- The mean incidence of adult idiopathic inflammatory myopathies in Salford, UK, is 17.6 (15.2–20.0)/1 000 000 person years.
- The incidence of adult idiopathic inflammatory myopathies increased significantly over the duration of the study.
- There is disagreement between EULAR/ACR classification criteria and expert opinion with regard to idiopathic inflammatory myopathy subtypes.

## Introduction

The idiopathic inflammatory myopathies (IIM) represent a spectrum of rare immune-mediated syndromes usually characterized by inflammation in skeletal muscle [1]. Studying the epidemiology of rare conditions can assist in the identification of risk factors, disease associations and temporal trends. Interrogation of differing geographically and genetically diverse populations can help to construct a more complete picture of underlying disease patterns.

A number of UK centres have contributed to national and international IIM research collaborations, but to date there has been no published report detailing the incidence or prevalence of adult IIM in the UK or to establish the relative proportion of the varying clinical subtypes. Moreover, previous international studies have focused on specific IIM subtypes, such as IBM or immune-mediated necrotizing myopathy (IMNM), are historic, were undertaken before recent developments in our understanding of the range of IIM subtypes and used widely varying methodologies and case acquisition strategies [2, 3].

The recently published combined EULAR/ACR classification criteria for adult and juvenile IIM represent potential progress in identifying IIM and various disease subtypes [4]. We present here the first epidemiological study to use these new criteria as part of disease verification. Briefly, the criteria comprise 16 variables, each with a corresponding weighted score. The gross sum of these scores can be used directly or converted into a probability (as a percentage) of an IIM diagnosis. The proposed cut-points for the diagnosis of a definite, probable and possible IIM are ≥90% (score ≥7.5 without biopsy; ≥8.7 with biopsy), 55–89% (5.5–7.4 without biopsy; 6.7–8.6 with biopsy) and 50–54% (score 5.3–5.4 without biopsy or 6.5–6.6 with biopsy), respectively. A second function is in the identification of one of four IIM disease subtypes, namely PM, DM, IBM or amyopathic DM (ADM).

The aim of this study was to identify all incident cases of IIM between 1 January 2007 and 31 December 31 2016 in the City of Salford, UK. A secondary aim was to characterize incident cases using clinical and laboratory data and, specifically, to compare the utility of the EULAR/ACR classification criteria against diagnosis made by expert opinion.

## Methods

This work represents part of a national quality improvement project aimed at identifying IIM cases for specialized disease commissioning, because accurate incidence data will inform future service planning. Given this context, approval for the conduct of the project was granted without a recommendation to seek more formal ethics authorization, in keeping with local policy.

### Denominator population

Salford is a City within Greater Manchester, UK, comprising a spectrum from densely populated areas to open rural space, whose mean annual population is publically available, stratified by age and sex. We excluded persons <18 years old in calculations of incidence. Salford Royal NHS Foundation Trust (SRFT) provides tertiary neuromuscular services for adults with an IIM in North West England, UK. SRFT is ideal to undertake an IIM epidemiological study as the sole provider of services for adult patients with IIM within our study area, with a longstanding status as a specialist neuromuscular service provider and with advanced information technology systems. It is routine practice locally for all cases of suspected IIM to be referred to the neuromuscular service even if, for example, they have predominantly or exclusively extramuscular IIM manifestations.

### Case definition

Adults diagnosed with IIM and resident in Salford between 1 January 2007 and 31 December 2016 were eligible. Using the EULAR/ACR classification criteria, all cases with definite IIM (≥90% probability, corresponding to a total aggregate score of ≥7.5 without muscle biopsy findings or ≥8.7 with biopsy data) were included, as were those with probable IIM (55–90% probability; aggregate score ≥5.5 without biopsy or ≥6.7 with biopsy) if the opinion of attending tertiary neuromuscular experts was of an IIM diagnosis [4]. The independent opinion on the certainty of an IIM diagnosis was obtained from at least two investigators in this instance. Cases were excluded if they were <18 years of age at disease onset, if their case did not meet probable criteria or if they met probable criteria but the consensus expert opinion was of a diagnosis other than IIM.

The EULAR/ACR criteria used with or without the decision tree identify the specific IIM subtypes of PM, DM, IBM and ADM or indicate if a case is unclassifiable, in which case the subtype determined by the decision tree would be PM by default unless a pattern of weakness suggestive of IBM is present. Biopsy data were always included where available. Although not standardized in formal criteria to the same extent, further IIM subtypes are well described and include the anti-synthetase syndrome (characterized by the presence of one of the eight described anti-tRNA-synthetase antibodies), IMNM (characterized by specific biopsy and/or autoantibody status) and overlap myositis (OM; where a specific associated connective-tissue disorder is present together with myositis, such as MCTD, SSc or RA) [1, 5]. In every case, the expert opinion of diagnostic subtype was recorded in addition to the subtype derived by the EULAR/ACR classification criteria.

### Case identification

Three stages of overlapping case ascertainment were used in SRFT. First, an ICD-10 coding search consistent with a recently published study was performed for all inpatient hospital episodes related to IIM at SRFT [6]. Second, all new patients referred to SRFT neuromuscular outpatient clinics were identified. Third, all persons enrolled in the UKMYONET database, a national research collaboration involving multiple centres around the UK, and enrolling IIM patients resident in Salford were identified. These data sets were merged, a manual review of all patient records was undertaken, and inclusion and exclusion criteria were then applied.

### Statistical methods

The mean adult population for each 1-year period was used as the denominator (each person then equal to one person year) for that respective year. Incidence rates were presented as numbers per million person years accompanied by 95% confidence intervals in parentheses. Statistical analyses were performed using SPSS v.23 (IBM Corp., Armonk, NY, USA) and MS Excel 2010 (V14.0; Microsoft Corp. Redmond, WA, USA). Categorical variables were presented as numbers and percentages, where appropriate. Descriptive, continuous variables with normal distribution were presented as a mean with the corresponding range and standard deviation. Statistical differences between groups for non-parametric variables were calculated using the Mann–Whitney *U* test and presented as exact *P*-values. Trend lines were presented with an accompanying coefficient of determination (*R*^2^).

## Results

The adult population of Salford grew steadily from 172 984 to 191 083 persons over the 10-year period between 2007 and 2016 (see supplementary data, available at *Rheumatology* online).

### Case ascertainment

The inpatient ICD-10 coding search resulted in identification of 922 cases. The search for new patients seen in rheumatology and neurology outpatient neuromuscular clinics identified 201 cases. Review of the UKMYONET database identified 33 cases resident in Salford at the time of diagnosis. After removal of duplicates, manual electronic record review and application of the inclusion and exclusion criteria, 32 incident cases of IIM were identified; 13/32 of these cases had biopsy data. The resident persons ultimately deemed not to have a diagnosis of IIM most commonly had inherited myopathies, such as a dystrophy or a metabolic myopathy, had episodes of rhabdomyolysis or had been incorrectly coded.

### Incidence

**Fig. 1:**
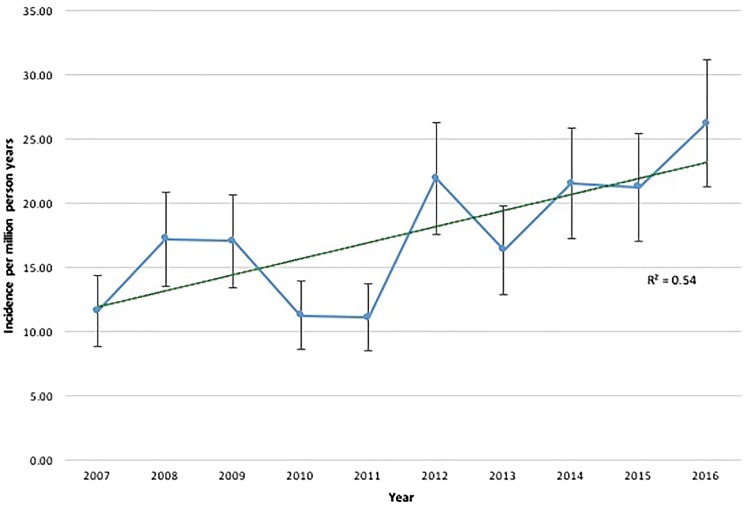
The annual incidence of idiopathic inflammatory myopathies in the City of Salford, UK Grey bars illustrate 95% confidence intervals; dashed green line illustrates linear trend line with accompanying coefficient of determination

The mean incidence of IIM in Salford throughout the 10-year study period was 17.6 (15.2–20.0)/1 000 000 person years. The incidence for females was higher than for males; 25.2 (21.7–27.8)/1 000 000 *vs* 10.0 (8.4–11.7)/1 000 000 person years, respectively. Stratifying cases by calendar year, there was a trend towards increasing annual incidence over the 10-year period (see Fig. 1). Thus, when comparing pooled data for 2007–2011 against 2012–2016, there was a clear and statistically significant increase in incidence over time [13.6 (11.5–15.7) *vs* 21.4 (18.3–24.5)/1 000 000 person years, respectively; *P* = 0.032].

### Clinical characteristics

**Fig. 2:**
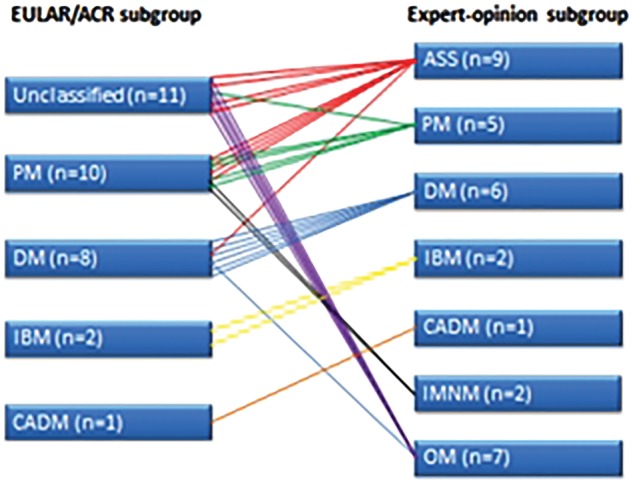
The relationship between EULAR/ACR classification criteria-assigned idiopathic inflammatory myopathy subtype and clinician-assigned idiopathic inflammatory myopathy subtype ADM: amyopathic DM; ASS: anti-synthetase syndrome; IMNM: immune-mediated necrotizing myopathy; OM: overlap myositis

Twenty-three of 32 incident cases were female. The mean age at disease onset was 58.1 years (21–81; s.d. 14.2). All but two cases had clinically apparent myositis, and the additional extramuscular IIM manifestations were broad. Ten of 32 had interstitial lung disease, 9/32 a rash consistent with DM, 6/32 an arthritis, 4/32 mechanic’s hands, 4/32 RP, and 3/32 had dysphagia. Three of 32 cases were associated with cancer; these were all associated with a DM subtype and were older than the remaining cohort (mean age 70.3 years). Using EULAR/ACR criteria, the largest subtype was PM (21/32), followed by DM (8/32), IBM (2/32) and ADM (1/32). When subtype was instead assigned by expert clinical opinion, the subtype frequencies for comparison were 5/32 PM, 6/32 DM, 2/32 IBM, 1/32 ADM, 9/32 anti-synthetase syndrome, 7/32 OM and 2/32 IMNM. Relevant patient-specific clinical information used in clinical subtyping is summarized in the supplementary data, available at *Rheumatology* online. Figure 2 illustrates how individual cases classified using EULAR/ACR criteria related to the clinically assigned subtype.

## Discussion

The overall adult IIM incidence of 17.6 (15.2–20.0)/1 000 000 person years sits within the range of estimates from other national geographical regions. A systematic review, summarizing the majority of these studies performed across five continents, estimated the incidence slightly higher than that described here at 20.0 (18.8–21.3)/1 000 000 person years [2]. However, few of the included studies used clinical or laboratory data to confirm the case diagnoses, and only 4/46 studies reported adult IIM subtype frequency. The last of these was published in 1999, therefore before the recent and rapid advances in nomenclature and subtype characterization [7–10]. In addition, the two largest studies reported the highest incidences [42.7 (40.9–44.4) and 66 (62–69)/1 000 000 person years, respectively], but identified cases solely on insurance database ICD-9 coding systems [11, 12]. There is considerable potential overlap in coding between IIM and other muscular disorders, such as inherited myopathies, dystrophies and metabolic myopathies, in addition to acquired pathologies, such as non-immune-mediated rhabdomyolysis, infections and alternative systemic inflammatory conditions. This is supported by our data, where our coding search identified 922 inpatient episodes but, after manual review, all but 32 cases were non-IIM. This systematic review thus concluded that coding searches alone without additional medical record review were likely to result in higher incidence and prevalence estimates, with the consequence of potentially skewing compound estimate figures. The present finding of a higher incidence of adult IIM in females is a consistent finding with all previous studies, except for some that focused on IBM and IMNM [2].

Two additional studies, published after the systematic review, add to the context of our results. The study of Svensson *et al.* [13] estimated an IIM incidence of 11 (10–12)/1 000 000 person years in a Swedish population. This relatively low estimate might have been affected by a single method of case ascertainment without manual record review, and because the denominator population was not restricted to the adult population alone. This study also focused an ICD-10 search on two diagnostic codes (M60.8 and M60.9) rather than the broader initial coding search performed in our study and other studies. Døbloug *et al.* [6] estimated an annual incidence of 6–10/1 000 000 person years in a Norwegian population, although this again did not report the incidence restricted to adults. This study also excluded diagnoses of IBM, ADM and OM, because these subtypes are not specifically identifiable by the Targoff or Bohan and Peter classifications [14, 15]. The relatively small number of incident cases in our own study might have influenced the overall estimate. However, whether the real incidence of IIM is higher in Salford than in other European populations is ultimately unclear.

Our data suggest an increasing incidence in adult IIM over the 10-year period studied. One previous study has demonstrated a statistically significant increase in IMNM incidence over time, with this trend suggested to be the result of a number of factors, including exposure to statin medications, linked to the anti-HMGCR-antibody-positive variant of IMNM [16]. Putative factors to explain the observed temporal increase in IIM incidence include improved awareness of IIM in the wider medical community and recognition of the extramuscular manifestations, which may present to a myriad of specialist services, and improved access to and performance of diagnostic procedures. That there might be a truly increasing IIM incidence should be borne in mind when future service development plans are being made.

The development of internationally accepted IIM classification criteria represents an important achievement. However, it is important to bear in mind that these criteria are likely to proceed through series of revision changes over time. In particular, the incorporation of more myositis-specific autoantibodies and IIM subtypes not at present specifically included now appears necessary. For instance, in our study, nearly 60% of the clinical subtypes indicated by EULAR/ACR classification would have been assigned differently by our clinical interpretation, perhaps a reminder that classification criteria are not designed primarily to be used in clinical practice. Of potential relevance to this is the relatively low number of cases (13/32) with biopsy data in our cohort. Given the invasive nature of the procedure, it is our typical practice to avoid biopsy to confirm IIM diagnoses in patients who already have a very high probability of the diagnosis based upon their clinical and other investigation findings. Our cohort contains a relatively high proportion of patients with anti-synthetase syndrome and OM (16/32), in whom we often feel biopsy is not necessary for this reason.

Although relatively small, this study benefits from a robust, multimodal case ascertainment process, blending methods from previous epidemiological studies. The study also uses new classification criteria for the first time in an IIM epidemiological study and allowed for detailed clinical phenotyping as a result of manual review of the medical records. Our study is limited by the population size, especially when considering the incidence of individual IIM subtypes. We chose to focus upon the City of Salford rather than, for example, the broader region of Greater Manchester, to ensure accurate case ascertainment and more complete data acquisition. There is always the potential for incomplete case acquisition in studies similar to ours, for example in those IIM cases with predominantly extramuscular manifestations not recognized to have an IIM and where the introduction of immunosuppression may prevent the evolution of the phenotype to include more characteristic IIM features. There is also the potential for patients who rapidly deteriorated with, for example, cancer-associated IIM or rapidly progressive interstitial lung disease and respiratory failure, to be missed. However, we believe that the routine local practice of referring all suspected IIM to the neuromuscular service, irrespective of predominant organ involvement, in addition to the structure of primary care referral pathways locally will have kept this to a minimum. Conversely, there is also the potential for the apparent increase over time to be partly or wholly attributable to a catch-up phenomenon, whereby cases that would have been missed 10 years ago are now being better identified, especially by non-neuromuscular unit colleagues, because of advances in diagnosis and local referral networks. Additionally, the clinical opinion on subtype, and therefore the relative performance of any classification criteria, is dependent on individual interpretation of the current literature, which may vary between clinicians.

### Conclusion

The mean incidence of adult IIM in Salford is 17.6 (15.2–20.0)/1 000 000 person years; it appears to be increasing over time and is in keeping with estimates from other international studies. There remains disparity between clinician-attributed IIM subtypes and those assigned by the recently developed EULAR/ACR classification criteria.

## Supplementary Material

Supplementary Data
