# Supplementary material for: Increasing incidence of adult idiopathic inflammatory myopathies in the City of Salford, UK: a 10-year epidemiological study
Source: Rheumatol Adv Pract. 2018 Sep 17;2(2):rky035. doi: 10.1093/rap/rky035 (PMC6649983; doi:10.1093/rap/rky035)
Supplement: Supplementary Data [file rky035_supp.docx]

# Supplementary Data

**Supplementary Table S1: ICD-10 code search terms used to screen all inpatient patient episodes over ten year study period to identify incident IIM**

| ICD-10 Code | Code descriptor |
| --- | --- |
| M33.1 | Other dermatomyositis |
| M33.2 | Polymyositis |
| M33.9 | Dermatopolymyositis, unspecified |
| M60.1 | Interstitial myositis |
| M60.8 | Other myositis |
| M60.9 | Myositis, unspecified |
| G72.4 | Inflammatory myopathy, not elsewhere classified |
| G72.8 | Other specified myopathies |
| G72.9 | Myopathy unspecified |
| G73.7 | Myopathy in other diseases specified elsewhere |

IIM: Idiopathic Inflammatory Myopathies

**Supplementary Table S2: The annual adult population of the City of Salford, UK**

| Population | 2007 | 2008 | 2009 | 2010 | 2011 | 2012 | 2013 | 2014 | 2015 | 2016 | Mean |
| --- | --- | --- | --- | --- | --- | --- | --- | --- | --- | --- | --- |
| Total | 172984 | 175090 | 176489 | 178686 | 180687 | 182708 | 183943 | 186109 | 188859 | 191083 | **181664** |
| Male | 85431 | 86558 | 87445 | 88647 | 89763 | 90820 | 91706 | 93049 | 94714 | 96020 | **90415** |
| Female | 87553 | 88532 | 89044 | 90039 | 90924 | 91888 | 92237 | 93060 | 94145 | 95063 | **91249** |

**Supplementary Table S3: Individual case characteristics**

| Age at onset/Gender | Antibody | Organ involvement | Relevant Investigations | Clinical Diagnosis | IIM probability | IMCCP Subtype |
| --- | --- | --- | --- | --- | --- | --- |
| 55/F | -Jo-1 | ILD, Myositis | Raised muscle enzymes, EMG and biopsy consistent | ASS | Definite | PM |
| 63/F | -Jo-1 | Arthritis, ILD, myositis | Raised muscle enzymes, EMG negative, biopsy myopathic (no specific IIM features) | ASS | Definite | PM |
| 44/F | -Jo-1 | Myositis, ILD, Raynauds, Mechanics hands | Raised muscle enzymes, EMG myopathic, biopsy consistent | ASS | Definite | PM |
| 58/F | N/A | Myositis | Raised muscle enzymes, EMG myopathic, biopsy endomysial T-cell infiltrate | PM | Definite | PM |
| 68/M | N/A | Myositis, DM rash | Raised muscle enzymes, EMG myopathic, no biopsy | DM | Definite | DM |
| 66/F | N/A | Myositis, IBM pattern of weakness | Raised muscle enzymes, EMG myopathic, biopsy diagnostic for IBM (Griggs definite^17^) | IBM | Definite | IBM |
| 60/F | N/A | Myositis | Raised muscle enzymes, EMG myopathic, biopsy PM features | PM | Definite | PM |
| 41/M | -Mi2 | Myositis, DM rash | Raised muscle enzymes, EMG myopathic, biopsy has DM features | DM | Definite | DM |
| 66/F | -CCP | Erosive polyarthritis, myositis | Raised muscle enzymes, EMG myopathic, no biopsy | OM | Definite | PM |
| 27/M | -U1-RNP | Myositis, dysphagia | Raised muscle enzymes, EMG myopathic, no biopsy | OM | Definite | PM |
| 76/M | N/A | Myositis and IBM pattern weakness | Raised muscle enzymes, EMG myopathic, biopsy diagnostic IBM (Griggs definite) | IBM | Definite | IBM |
| 81/F | -PL7, -Ro52 | Myositis, DM rash, abnormal capillaroscopy | Raised muscle enzymes, EMG myopathic, no biopsy | ASS | Definite | DM |
| 47/F | -Jo1, -Ro52 | Myositis, ILD, myopericarditis | Raised muscle enzymes, no EMG, no biopsy | ASS | Definite | PM |
| 21/F | -PmScl, -Ku | Myositis, Gottrons papules, arthritis, SSc features | Raised muscle enzymes, no EMG, biopsy DM features | OM | Definite | DM |
| 63/F | -Mi2 | Myositis, DM rash, abnormal capillaroscopy | Raised muscle enzymes, EMG myopathic, biopsy DM features | DM | Definite | DM |
| 72/M | N/A | Myositis, DM rash, Lymphoma | Raised muscle enzymes, EMG myopathic, biopsy mild non-specific IIM features | DM | Definite | DM |
| 63/F | N/A | Mild myositis, ILD, Limited SSc | Raised muscle enzymes, No EMG, No biopsy | OM | Definite | PM |
| 63/F | -U1-RNP | Myositis, ILD | Raised muscle enzymes, No EMG, no biopsy | OM | Definite | PM |
| 55/M | -SAE | DM rash, no myositis/weakness | Normal muscle enzymes, no EMG, no biopsy | ADM | Definite | ADM |
| 55/M | -Jo1, -Ro52 | No weakness/ myositis, ILD, Raynauds, arthritis, mechanics hands | Normal muscle enzymes, no EMG, no biopsy | ASS | Possible | PM |
| 66/M | -PmScl | Myositis, Raynauds, ILD | Raised muscle enzymes, EMG myopathic, biopsy PM features | PM | Definite | PM |
| 71/F | -Jo1, -Ro52 | Myositis, ILD, Raynauds, arthritis | Raised muscle enzymes, no EMG, no biopsy | ASS | Definite | PM |
| 59/F | N/A | Myositis | Raised muscle enzymes, EMG myopathic, biopsy PM features | PM | Definite | PM |
| 66/F | N/A | Myositis | Raised muscle enzymes, no EMG, no biopsy | PM | Definite | PM |
| 71/F | -SRP | Myositis | Raised muscle enzymes, no EMG, biopsy myopathic (no IIM features) | IMNM | Probable | PM |
| 50/M | N/a | Myositis, Dysphagia | Raised muscle enzymes, EMG myopathic, biopsy scant CD8+ infiltrate, necrosis | IMNM | Definite | PM |
| 65/F | -TIF1g | Myositis, DM rash, dysphagia. Myelodysplasia | Raised muscle enzymes, EMG myopathic, no biopsy | DM | Definite | DM |
| 46/F | -Jo1, -Ro52 | Myositis, ILD, arthritis, mechanics hands | Raised muscle enzymes, EMG normal, biopsy consistent | ASS | Definite | PM |
| 53/F | -Jo1 | Myositis, ILD, mechanics hands | Raised muscle enzymes, no EMG/biopsy | ASS | Definite | PM |
| 66/F | -dsDNA | Myositis, membranous nephropathy, lupus rash | Raised muscle enzymes, no EMG/biopsy | OM | Definite | PM |
| 29/F | -centro-  mere | Myositis but no weakness, limited SSc | Raised muscle enzymes, no EMG/biopsy | OM | Possible | PM |
| 74/F | -NXP2 | Myositis, DM rash, Cancer | Raised muscle enzymes, no EMG, no biopsy | DM | Definite | DM |

Biopsy interpretation in keeping with European Neuromuscular Centre criteria (ENMC) ^18^ ^19^. ILD: Interstitial Lung Disease; IBM: Inclusion Body Myositis; EMG: ElectromyogramASS: anti-synthetase syndrome; OM: Overlap myositis; ADM: amyopathic dermatomyositis; IMNM: immune-mediated necrotising myopathy.
